# SSMD-UNet: semi-supervised multi-task decoders network for diabetic retinopathy segmentation

**DOI:** 10.1038/s41598-023-36311-0

**Published:** 2023-06-05

**Authors:** Zahid Ullah, Muhammad Usman, Siddique Latif, Asifullah Khan, Jeonghwan Gwak

**Affiliations:** 1 Department of Software, Korea National University of Transportation, Chungju, 27469 South Korea; 2 Department of Computer Science and Engineering, Seoul National University, Seoul, 08826 South Korea; 3 Faculty of Health and Computing, University of Southern Queensland, Toowoomba, QL 4300 Australia; 4 Pattern Recognition Lab, DCIS, PIEAS, Nilore, Islamabad, 45650 Pakistan; 5 Department of Biomedical Engineering, Korea National University of Transportation, Chungju, 27469 South Korea; 6 Department of AI Robotics Engineering, Korea National University of Transportation, Chungju, 27469 South Korea; 7 Department of IT Energy Convergence (BK21 FOUR), Korea National University of Transportation, Chungju, 27469 South Korea

**Keywords:** Diseases, Eye diseases, Computational biology and bioinformatics, Image processing

## Abstract

Diabetic retinopathy (DR) is a diabetes complication that can cause vision loss among patients due to damage to blood vessels in the retina. Early retinal screening can avoid the severe consequences of DR and enable timely treatment. Nowadays, researchers are trying to develop automated deep learning-based DR segmentation tools using retinal fundus images to help Ophthalmologists with DR screening and early diagnosis. However, recent studies are unable to design accurate models due to the unavailability of larger training data with consistent and fine-grained annotations. To address this problem, we propose a semi-supervised multitask learning approach that exploits widely available unlabelled data (i.e., Kaggle-EyePACS) to improve DR segmentation performance. The proposed model consists of novel multi-decoder architecture and involves both unsupervised and supervised learning phases. The model is trained for the unsupervised auxiliary task to effectively learn from additional unlabelled data and improve the performance of the primary task of DR segmentation. The proposed technique is rigorously evaluated on two publicly available datasets (i.e., FGADR and IDRiD) and results show that the proposed technique not only outperforms existing state-of-the-art techniques but also exhibits improved generalisation and robustness for cross-data evaluation.

## Introduction

Diabetic retinopathy (DR) is an eye condition that can leads to vision loss or blindness in people with diabetes. It is mainly caused by the damage to the blood vessels of the retina^1^. In diabetic patients, the excessive growth of glucose in the blood affects retinas, which is the innermost layer of the eye. It processes visual information by transferring the light through neural signals and coordinating with the brain. The retina receives blood nourishment like all parts of the human body through the micro blood vessels. The blood sugar level with the uninterrupted blood flow must be retained^2^. The high blood sugar level may damage the tiny blood vessels even in the prediabetes stage. Over time, blood vessels in the retina start leaking fluid that causes swelling and blur vision in DR patients. According to the World Health Organization (WHO), there were about 463 million diabetes patients in the year 2019 globally and more than 77% of them suffer from DR^3^. With each passing year, the prevalence of DR is increasing, which can lead to a higher number of patients with vision problems or even blindness. Hence, early diagnosis of DR is important through regular screening for preventing further complications.

**Figure 1:**
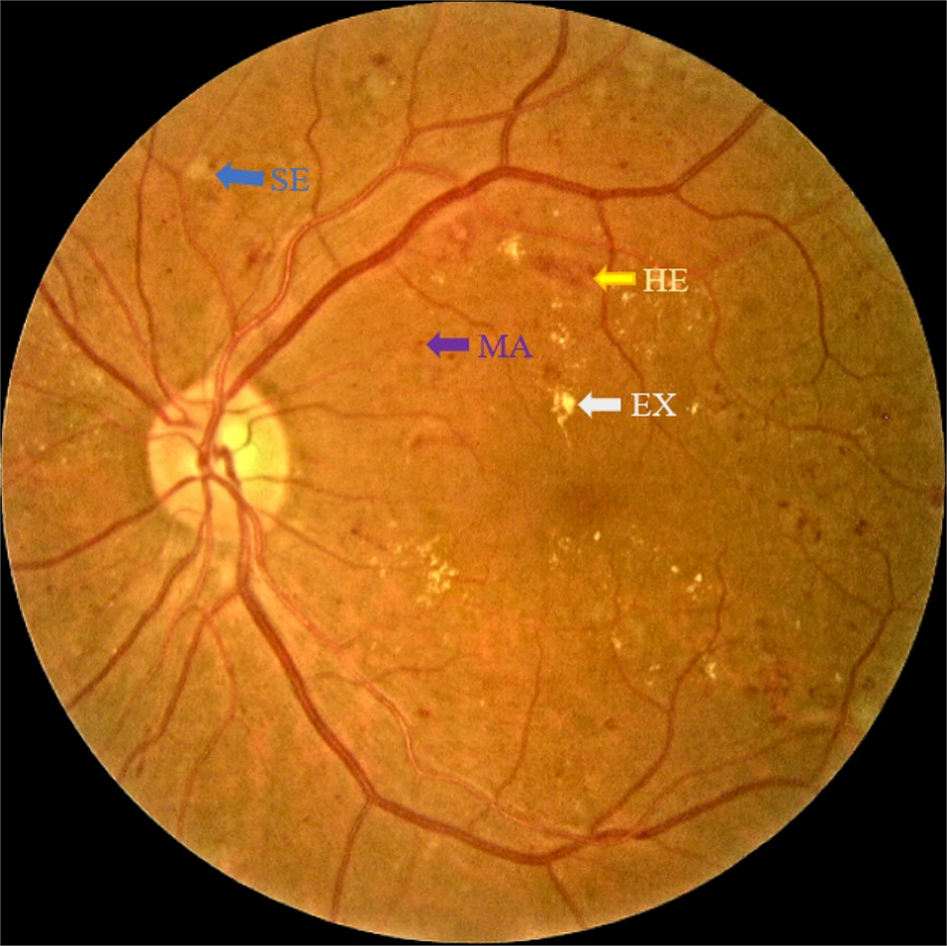
Illustration of fundus image with characteristics of DR lesion. *EX* hard exudate, *MA* microaneurysm, *HE* hemorrhage, *SE* soft exudate.

A fundus photography is usually used to screen DR and other eye related illnesses^4^. A fundus image visualizes the details of entire layers on retina which enables a doctor to provide the most accurate diagnosis. Figure 1 presents an example of a DR retina by fundus photography, which consists of multiple lesions such as hemorrhage (HEs), microaneurysm (MAs), hard exudates (EXs), and soft exudates (SEs). Among them, MA is the earliest clinically visible evidence of DR, which appears as small red dots. In addition to MA, moderate non-proliferative DR contains ‘blot’ or ‘dot’ shaped hemorrhages (HEs). EXs are vivid yellow-white intra-retinal deposits that can vary from small specs to larger patches. SEs are greyish-white patches of discoloration in the nerve fiber layer, which usually appear in the severe DR stage.

Early detection of DR using automated systems is highly desired. Unfortunately, the current DR detection practices are unable to provide the fully automated services. Specifically, these practices need a well-trained clinician to manually examine digital color fundus images of the retina and identify the DR disease by locating the lesions (i.e. MAs, HEs, EXs, and SEs). Although such practice can provide accurate detection, however, this task is hectic, time-consuming and fully depends on the expertise of well-trained practitioners. Therefore, considerable efforts are being made to develop computer-aided DR diagnosis tools for screening patients as well as to efficiently facilitate the ophthalmologists^4^. Such automated tools will be able to assist clinicians in identifications, screening, and monitoring of the disease for accurate and ultimate measurements of retinal lesions.

Various studies attempted to automate the screening of DR^4^. However, due to: (1) the noisy images (impulsive noise, bright border reflections, optical reflections), (2) structure complexity (size, shape, intensity), and (3) appearance of non-lesion structures (vessel reflections, drusen, nerve fiber reflections), it is very challenging. Researchers also explored deep learning (DL) techniques to solve this problem^5^, however, the unavailability of larger training data is the major road block towards the development of an accurate and robust solution. We propose to use semi-supervised model to address this issue. Semi-supervised learning based systems offer opportunities to learn from both labeled data and unlabeled data. Such solutions showed improved results in various applications including knee osteoarthritis severity grading^6^, speech emotion recognition^7^, and Covid-19 detection^8^. However, to the best of our knowledge, none of the study use multitask learning semi-supervised model for diabetic retinopathy segmentation.

In this study, we propose a semi-supervised multitask learning approach that enables us to exploit the larger unlabeled data in the unsupervised training phase to improve the performance. Specifically, we propose a novel architecture namely semi-supervised multi-task Decoders-UNet (SSMD-UNet), which consists of one encoder and four decoder branches, among them one is considered as a primary decoder and the rest are considered as auxiliary decoders. The proposed SSMD-UNet is trained in two phases i.e., unsupervised and supervised phase. In the unsupervised phase additional abundantly available data is utilized for training of an auxiliary task. Specifically, SSMD-UNet is trained for the reconstruction task during which the encoder branch learns to produce the optimal latent representation. During the supervised phase, the network is trained in multitask learning to identify one of the lesions (e.g., HE) as the primary task. We consider the detection of the other three lesions (e.g., MAs, EXs, and SEs) and reconstruction are considered as auxiliary tasks. We trained four separate models; each of these is optimized for one disease detection. The propose scheme has been rigorously evaluated and analysed on two publicly available datasets (i.e., FGADR^5^ and IDRiD^9^). The results show that the proposed scheme outperforms the existing state-of-the-art techniques and demonstrates the significant robustness as well.

## Related work

Over the past few years, various studies attempt to solve the problems of DR lesion detection and segmentation, and highlight the challenges^10^. In particular, deep learning based methods achieve significantly better performance^11^. The DR detection/segmentation research is mainly categorized into two groups: traditional machine learning (ML) based approaches and modern DL-based approaches. The traditional methods use fundus images to automatically detect one or several pre-selected DR-related lesions^12^, such as EXs, HEs, and MAs. A typical segmentation methods consist of region growing methods to devise various image regions based on some uniformity criteria such as color and gray level^12^, mathematical morphology operations performed by evaluating geometrical structures of retina components^12^. Traditional methods are usually based on handcrafted features (e.g., local binary pattern (LBP)^13^, intensity difference and gradient^14^ etc.) and learning-based features obtained from raw image data by learning latent features, discriminative representation using ML techniques^15^. Unfortunately, the classical techniques are unable to model the complex structure in fundus images and have issues of scalability.

In contrast, DL based approaches can learn more complex structures and becoming very popular in DR detection/segmentation^5,16^. DL techniques ensure to simultaneously learn and understand higher-level and lower-level representation from the input images without requiring the handcrafted features^17^. These characteristics making the DL-based techniques to emerge as an effective tool to reshape the medical image analysis for healthcare applications^17^. In the medical image analysis field, the convolutional neural networks are very famous among other DL techniques^18^. The existing literature consists of different configurations and variants of CNN’s in which AlexNet^19^, ResNet^20^, and VGG^21^, are the most popular.

In retinal image analysis, DL has been widely employed due to its unique characteristics of preserving local image relations. For instance, Chudzik et al.^22^ applied a fully convolutional neural network with the batch normalization layers and the dice coefficient loss function to detect and segment MAs. They have evaluated their proposed model on E-Ophtha^23^ and achieve 0.84% sensitivity rate. Mo et al.^24^ presented an image-level fully convolutional residual network for EX segmentation. Their proposed model is capable of producing a probability map of EX for fundus image using only one forward pass. Tan et al.^25^ presented CNN-based model to segment multiple lesions including EX, MA, and HE, simultaneously. This work demonstrated that it is possible to simultaneously segment several lesions using a single CNN architecture. They have evaluated their proposed methodology on CLEOPATRA database^25^ which consists of 298 images and achieved 0.87%, 0.62%, and 0.46%, sensitivity rates for EXs, HEs, and MAs, respectively. Gwenole et al.^4^ presented a novel technique using CNN to detect referable DR and automatically segment DR lesions. They have created heatmaps of the convolutional layer that leads to explore new biomarkers in images and achieve improved performance. The heat map attained a similar accuracy for lesions like a pixel-wise trained convolution network. Various other studies^26,27^ also presented similar architecture to segment DR lesions. However, most of these studies evaluated their model using single datasets without considering to evaluate the generalisation of their proposed frameworks.

Aziz et al.^28^ proposed a novel methodology for hemorrhage detection. First, they enhanced the quality of the image, using contrast limited adaptive histogram equalization to improve the contrast of an image. Then the gamma correction is utilized to adjust the brightness level. Furthermore, the seed points extraction technique is employed to detect HEs. They have validated their methodology using DIARETDB1^29^ and DIARETDB0^30^ and obtained promising results. Wang et al.^31^ segmented the DR lesion by implementing a contextual net and achieved high accuracy. In contextual net, they incorporated supervision features to avoid overfitting. This contextual supervision model performance is analyzed through the fundus database where they reported the exact prediction but poor severity classification. Manisha and Susan^32^ have carried out DR detection, classification, and segmentation tasks. They reported that the pre-trained model i.e., DenseNet121 is the most suitable model for DR image classification. Whereas, EfficientNet-B0 and MobileNetV1 are effective for DR detection. In the DR segmentation task, PSPNet with focal loss provides efficient results and outperforms the pre-trained networks. Liu et al.^33^ segmented EXs by proposing a dual-branch network with dual-sampling modulated dice loss. This network utilizes two branches with partial weights sharing to learn representations and classifiers for EXs in various sizes. They compared their proposed model with five well-known deep learning-based methods: Unet++^34^, DoubleUnet^35^, SPNet^36^, DNL^37^, and Deeplabv3+^38^, and achieved better results than these five models. Huang et al.^39^ proposed a global transformer block and a relation transformer block for incorporating attention mechanisms and preserving detailed information for DR segmentation. The model has been evaluated on IDRiD^9^ and DDR^40^ datasets and achieves reasonable results.

Recently, MTL techniques are getting popular in DR segmentation due to their improved generalisation power. In MTL, models are developed to learn generalised representations by solving multiple related tasks together^41^. Yang et al.^42^ presented a hybrid segmentation method for vessel segmentation which is a combination of image fusion network and multitask (MT) segmentation network. The MT segmentation network segment the thin vessels and thick vessels separately from fundus images using U-Net. The model is evaluated using three publicly available datasets such as, CHASE_DB1^43^, DRIVE^44^, and STARE dataset^45^, and attained improved performance on these datasets. Zhao et al.^46^ proposed a *W*-net to segment the optic disc (OD) and the exudates simultaneously in retinal images using the MTL scheme. They have evaluated their proposed model on two publicly available datasets such as e_ophtha_EX (i.e., comprised of 82 fundus images) and DiaRetDb1 (comprised of 89 fundus images) datasets and obtained 94.76% and 95.73% F1-score for OD segmentation, and 92.80% and 94.14%, for EXs segmentation. Clement et al.^47^ proposed a multi-task CNN architecture to segment red lesion and bright lesions in fundus images. They have improved the segmentation accuracy of the retinal lesion by using image-level annotation. The model is evaluated using four different datasets, such as DIARETDB1^29^, IDRID^9^, e-optha exudate^23^, and EyePACS^26^ and obtained improved results. Most of the above studies utilise MTL learning in supervised setting without exploiting the abundantly available unlabelled data to improve the performance. In particular, we present a semi-supervised MTL method that can learn generalised representation and effectively exploit unlabelled data compared to the semi-supervised techniques^48,49^ in DR segmentation.

## Proposed method

We propose an MTL based framework which incorporates the semi-supervised learning by using a single encoder and five decoder branches. By using five decoder branches, the model is able to learn generalised representations by performing multiple tasks (i.e., segmentation and reconstruction) simultaneously. We consider the segmentation of one disease among four (MAs, HEs, EXs, and SEs) as the primary task, and the segmentation of the remaining three diseases along with the reconstruction task is considered as auxiliary tasks. Incorporation of reconstruction task enables the model to exploit the unlabelled dataset during the unsupervised phase, in which a single branch (i.e., reconstruction) of a decoder is optimized and the model acts like a conventional autoencoder network.

Our proposed model is motivated by semisupervised multi-task learning. Here we are utilizing multiple decoders to learn shared representations that help improve the generalization and performance of the system. In addition, it also enables us to utilize the additional abundantly available unlabelled data in the training pipeline of the system. This also helps improve the generalization and performance of the proposed system. We empirically evaluated the model and showed the benefits of using additional data, robustness analysis, and the effect of auxiliary tasks in “Results and discussion”.

**Figure 2:**
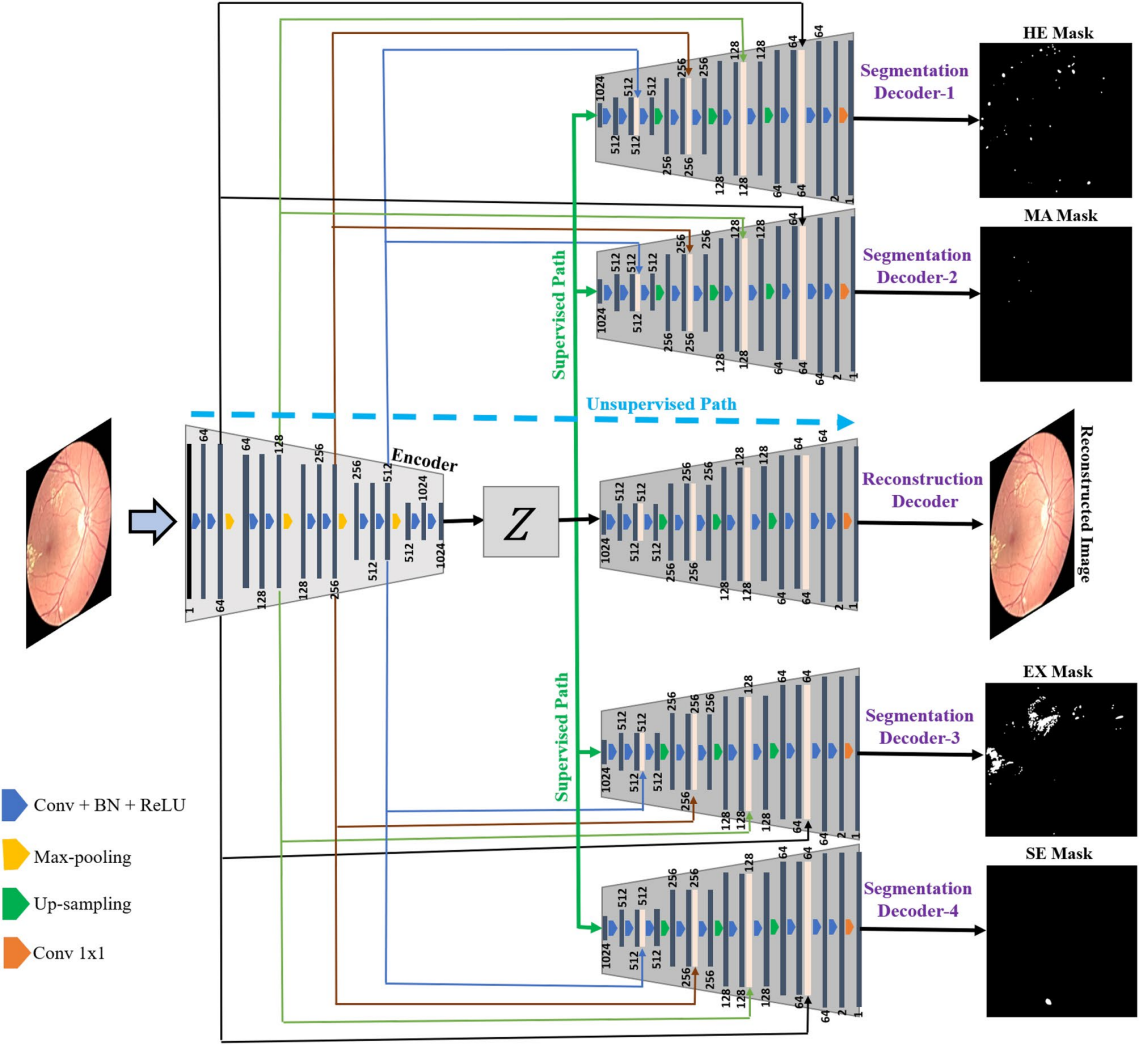
Proposed SSMD-UNet framework, where each decoder is designed for predicting one annotations, and one decoder is reconstructing the image. The dotted blue line exhibit the unsupervised path and the green line exhibit supervised path.

Figure 2 demonstrates the proposed model architecture which consists of single encoder and five decoder branches. One decoder branch performs reconstruction and the other four perform the segmentation of each disease. To further elaborate the model details, we divide the model into two parts based on the tasks (i.e., segmentation and reconstruction). The proceeding subsection describes both parts of proposed model.

### Pre-processing

**Figure 3:**
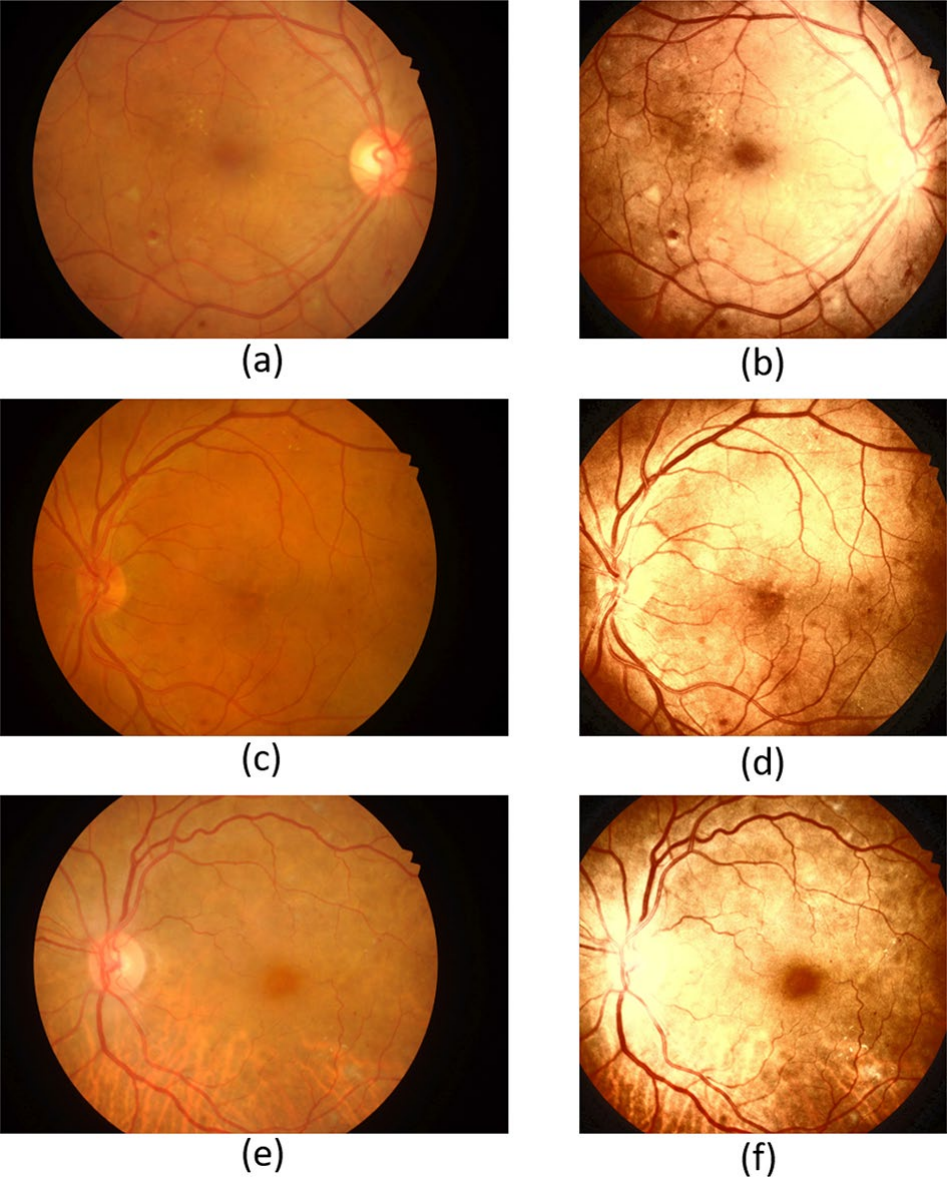
Fundus photograph preprocessing. Original images (**a**,**c**,**e**) are transformed into (**b**,**d**,**f**).

The DR lesions detection from fundus images is a challenging and important task. Due to masking on DR lesions, the images taken with digital imaging devices have various reflections and shadows. Effects such as some tinted lesions, bubble appearances, uneven lighting, noise, and specular reflections are part of the fundus images. Likewise, the selected datasets have prolific intensity as well as dimension variation. Hence, we apply a pre-processing step to improve the quality of training data as shown in Fig. 3. We first cropped the images from EyePACS and IDRiD^9^ datasets to remove the blank areas from all sides and then applied histogram equalisation^50^. Finally, we used a bicubic interpolation to resize the images from all the datasets to $512 \times 512$ based on their aspect ratio and normalised the intensity values.

### Segmentation task

As depicted in Fig. 2, the proposed model consists of four decoder branches that perform the segmentation of each disease (i.e., MAs, HEs, EXs, and SEs). The architecture of all decoder branches are identical and are inspired by the conventional UNet architecture^51^. Our UNet^51^ is based on convolutions neural networks (CNNs), which consists of a contractive (encoder/down), bottleneck (middle bottom), and upsampling (expansion) phase. The contractive part is comprised of a rectified linear unit (ReLU) placed after every second convolution layer, further, using the max-pooling layer, the result is then downsampled. This contraction increases feature information and reduces the spatial information. The expansive pathway combines the spatial information and feature information through a sequence of up-convolutions and concatenations with high-resolution features from the contracting path. We have employed five identical decoders in the proposed architecture that dilates the information at various levels by integrating the features learned at the corresponding level of the encoder branch through the residual connections. Finally, each decoder network learns to localize one disease for which it has been optimized.

### Input reconstruction task

The proposed MTL architecture also includes one unsupervised reconstruction branch (as shown in Fig. 2), which works like a standard autoencoder (AE) during training. AE mainly trained in an unsupervised way to learn comprised features by performing the reconstruction. In a typical AE, encoder part takes an image as input to encode into a compressed latent features, while the decoder is tasked to reconstruct the input image from compressed representation. In our framework, AE encode an input vector $x \in$ ${\mathbb {R}}^{I}$, this input is linearly mapped by the encoder with a set of weights $W^{1}_{e}$ $\in$ ${\mathbb {R}}^{K_1 \times I}$ with $K_1$ units. Then, added a bias vector $b^{1}_{e}\in$ ${\mathbb {R}}^{K_1}$ and applied a nonlinear activation function $f_e$ to generate the first layer outputs $h_1$ = $f_e(W^{1}_{e} \cdot x + b^1_e) \in {\mathbb {R}}^{K_1}$. The next layer outputs such as, $h_2$ = $f_e(W^{2}_{e}\cdot h_{1} + b^2_e) \in {\mathbb {R}}^{K_2}$ can be computed using the prior outputs and so on, until the final representation is computed such as *z* = $f_e(W^{L}_{e} . h_{L-1} + b^L_e) \in {\mathbb {R}}^{K_L}$ for a network with *l* layers.

To obtain the reconstructed input ${\hat{x}} \in \mathbb {R^I}$, the decoder maps the encoded representation *z* with another set of weights $W^{L}_{d}$ $\in$ ${\mathbb {R}}^{K_{L-1} \times K_L}$ as ${\hat{h}}_{L-1}$ = $f_d(W^{L}_{d} \cdot {z} + b^L_d) \in {\mathbb {R}}^{K_{L-1}}$ and so on until the final reconstruction ${\hat{x}} = f_d(W^{1}_{d} \cdot {\hat{h}}_{1} + b^1_d) \in \mathbb {R^I}$. The term $f_d$ is decoding activation function, and $b^{l}_{d}$ and $W^{l}_{d}$ are respectively the decoding bias and the weights matrix of layer *l*. AE in its original form, learn features by reducing the error between the input *x* and its decoded version ${\hat{x}}$. During the learning process, the cost function commonly used for optimization is the mean square error (MSE)^52^, which can be defined as follows:

1 $${\mathcal {L}}_{Rec}(x,D_{\delta }(E_{\theta }(x)))=\Vert {x-\hat{x}}\Vert _{2}^{2}$$

### Multitask training scheme

The proposed architecture exploits MTL to optimise the performance for primary tasks which is the localization of the lesion. There is a total of five tasks for which each decoder is assigned, specifically, four supervised (i.e., segmentation) tasks and one unsupervised (i.e., reconstruction) task. Among four supervised segmentation tasks, only one is considered as the primary task during training and the rest are trained as auxiliary tasks along with the unsupervised reconstruction task. In Eq. (2), we present the SSMD-UNet loss ${\mathcal {L}}_{\text {SSMD-UNet}}$ as a function of supervised and unsupervised losses.

2 $$\begin{aligned} {\mathcal {L}}_{\text {SSMD-UNet}}= & {} \alpha *{\mathcal {L}}_{\text {Rec}}+{\mathcal {L}}_{Seg}, \end{aligned}$$

3 $$\begin{aligned} {\mathcal {L}}_{Seg}= & {} \beta *{\mathcal {L}}_{Seg^{1}}+(1-\beta )*\big ({\mathcal {L}}_{Seg^{2}}+{\mathcal {L}}_{Seg^{3}}+{\mathcal {L}}_{Seg^{4}}\big ). \end{aligned}$$

Here, ${\mathcal {L}}_{\text {Rec}}$ is the reconstruction loss of the reconstruction branch (defined in Eq. 1), ${\mathcal {L}}_{Seg^{1}}$, ${\mathcal {L}}_{Seg^{2}}$, ${\mathcal {L}}_{Seg^{3}}$, and ${\mathcal {L}}_{Seg^{4}}$ are losses for the four segmentation tasks (i.e., HE, MA, EX and SE localisation). Here ${\mathcal {L}}_{Seg^{1}}$ is considered as the loss of primary task while ${\mathcal {L}}_{Seg^{2}}$, ${\mathcal {L}}_{Seg^{3}}$, and ${\mathcal {L}}_{Seg^{4}}$ denote the losses of auxiliary tasks ; $\alpha$ and $\beta$ are the trade-off parameters to control the weight of each loss term.

For a given input, i.e., in Decoder-1, we focus to solve HE as the primary task therefore, we use ${\mathcal {L}}_{Seg^{1}}$ for this. ${\mathcal {L}}_{Seg^{1}}$ is the loss of the primary tasks. This is the beauty of our proposed model for a given input, we can train the model for one primary task by giving more weights $\beta$ and penalizing the auxiliary task. The model will focus on accurately detecting primary tasks and also segmenting the auxiliary task as a byproduct. This mainly depends on the problem that we want to solve.

For the input data *x*, the overall model is trained in two phases: (1) the unsupervised (reconstruction) phase and (2) the supervised (segmentation) phase. In the unsupervised learning (reconstruction) phase, the model updates the encoder ($E_{\theta }$) and the reconstruction decoder ($D_{Rec}$) and minimises the reconstruction error (defined in Eq. 1) by encoding *x* into latent representation *z* and reconstructing the $\hat{x}$.

In the supervised learning phase, the encoder ($E_{\theta }$) and the segmentation decoders ($D_{Seg^k}$) are updated to minimise the segmentation error. We employ dice score loss for the optimisation of segmentation tasks which can be defined as below:

4 $$\begin{aligned} \mathcal {L_{Seg^{k}}}(x,D_{Seg^k}(E_{\theta }(x))) = (1 - \frac{2\times S_{pred}\cap S_{gt}}{S_{pred} \, \cup \, S_{gt}}), \, \end{aligned}$$

where $\,k \,\epsilon \, \{1,2,3,4\}$, while, $S_{pred}$ and $S_{gt}$ denote the predicted and ground truth segmentation, respectively.

## Experimental setup

### Datasets

#### FGADR dataset

**Figure 4:**
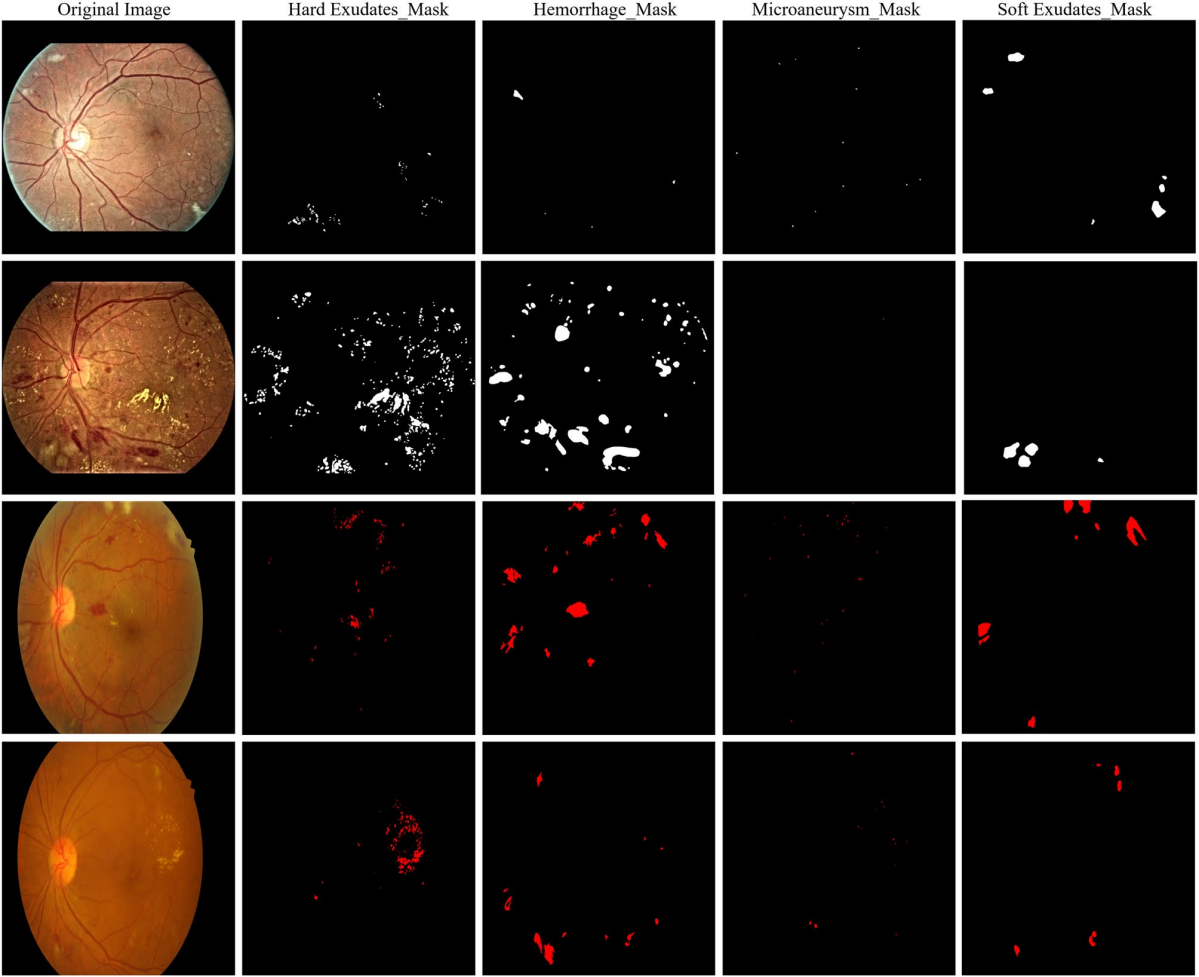
First and second rows are the illustration of fundus images from the FGADR dataset with annotated characteristics of DR lesion, whereas the third and fourth rows are from the IDRiD dataset with annotated characteristics of DR lesion.

The fine-grained annotated diabetic retinopathy (FGADR)^5^ dataset comprised of two sets. Seg-set and Grade-set. The Seg-set is made available from the corresponding author on reasonable request, the dataset consists of 1842 images with fine-grained pixel-level lesion annotations. The lesions consist of HEs, MAs, SEs, EXs, NV, and IRMA. During experimentation, we follow the data usage agreement provided by Zhou et al.^5^ and all the experiments were carried out in accordance with relevant guidelines and regulations. It is noticeable that the FGADR dataset consists of six lesions, each having its masks. We used NV and IRMA as an unlabeled data as they have less samples with ground truth i.e., 49 and 159 masks, respectively. Whereas, HE, MA, SE, and EX comprised of 1842 masks each. Figure 4 shows an example of fundus images and their annotated regions from the FGADR^5^ and IDRiD^9^ datasets, whereas the EyePACS dataset is unannotated dataset.

#### IDRiD

The Indian Diabetic Retinopathy Image Dataset (IDRiD dataset) is publicly available and can be downloaded from IEEE Dataport Repository^9^, under a Creative Common Attribution 4.0 license. More detail information about the data is available in the data descriptor^9^. We follow the data usage agreement provided by Porwal et al.^9^.

The IDRiD^9^ dataset consists of fundus images captured during real clinical examinations in an eye clinic in india using Kowa VX fundus camera. The obtained images have 50 degree field of view with a resolution of 4288 $\times$ 2848. The images are separated into three parts, corresponding to three different learning tasks and accompanied by the respective types of ground truth. The first part is designed for the development of segmentation algorithms that comprised of 81 images (54 train set and 27 test set) with pixel level annotations of DR lesions (MAs, HEs, EXs, SEs) and the optical disk. The second part corresponds to a DR grading task and contains 516 images divided into train set (413 images) and test set (103 images) with DR and Diabetic Macular Edema (DME) severity grades. Finally, the third part corresponds to a localization task and contains 516 images with the pixel coordinates of the optic disk center and fovea center (again split in a 413 train and 103 test set). Using this dataset, we only consider the pixel level annotated images (i.e., 81) to evaluate our SSMD-UNet.

#### Kaggle-EyePACS

The Kaggle-EyePACS dataset is publicly available dataset^26^. The Kaggle diabetic retinopathy detection challenge dataset consists of 35,126 training samples and 53,576 testing samples. A clinician has graded all images according to the International Clinical Diabetic Retinopathy Disease scale. The images are collected from different sources with various lighting conditions and weak annotation quality. The presence of DR in each image is rated on a scale of 0–4. In this dataset, some images contain artifacts, and are out of focus, underexposed, or overexposed. We followed the data usage agreement provided by EyePACS.

## Data availability and usage statement

All the above mentioned datasets are publicly available except FGADR that is available on request for research purposes. The Kaggle-EyePACS and IDRiD datasets utilized in this study were downloaded from publicly available sources. The Fine-Grained Annotated Diabetic Retinopathy (FGADR) datasets used during the current study available from the corresponding author on reasonable request^5^. We confirm that all the experiments were carried out in accordance with relevant guidelines and regulations. As all datasets used in this work are public, therefore, we followed the protocols mentioned by the data releasing organisations in their respective licenses.

### Training strategy

**Figure 5:**
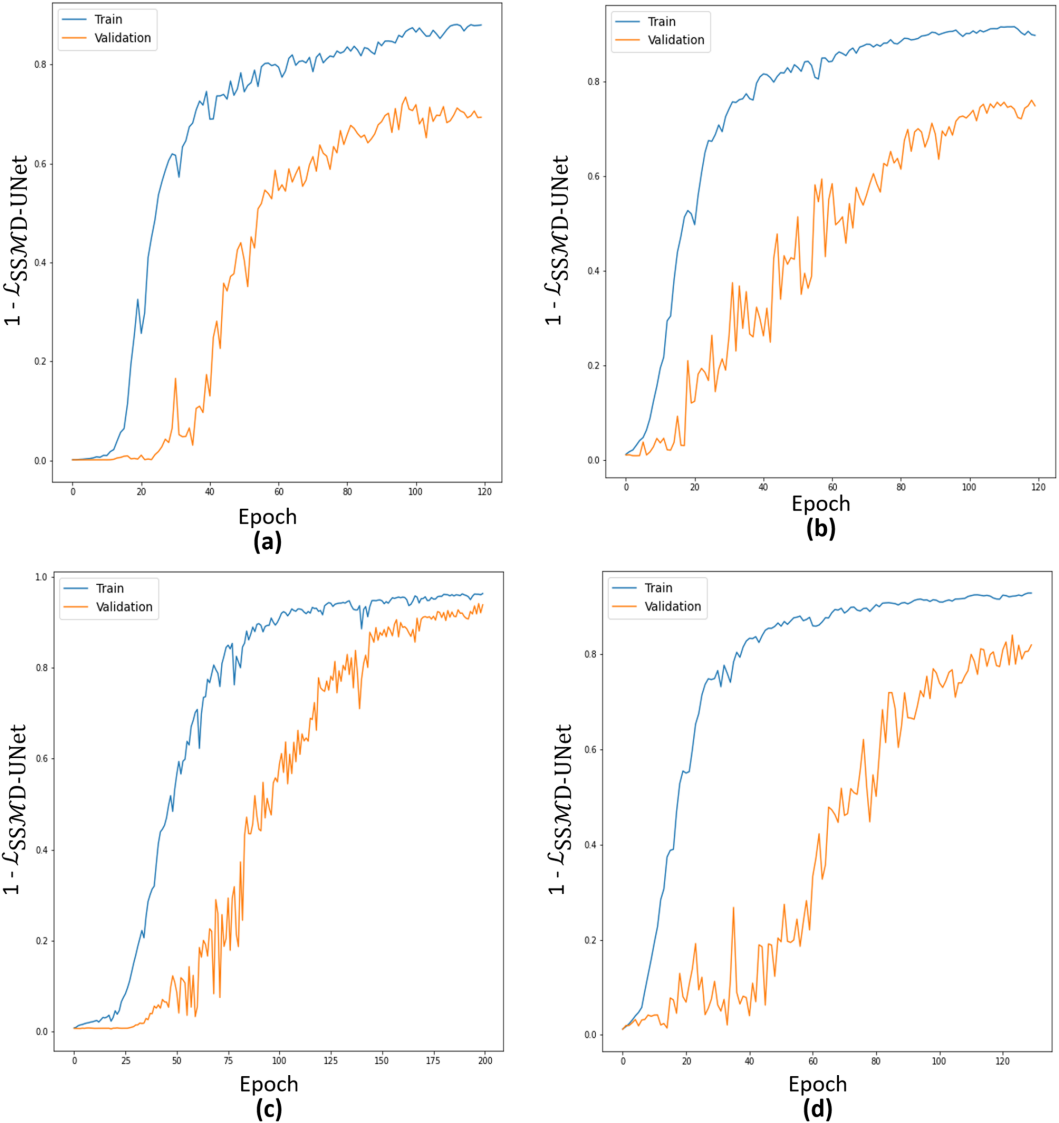
Learning curves of models trained for the segmentation of EX, MA, SE, and HE are shown in (**a–d**), respectively.

The step-by-step training strategy of our semi-supervised architecture is illustrated in Fig. 2. The single encoder part is comprised of five convolutional layers where a max-pooling layer is following each convolutional layer. These convolutional layers find out the main regions within the fundus image and create feature maps. We initialize the model randomly and then train the unsupervised path of the model. In particular, at first hand, we train the SSMD-UNet using unlabelled data such as EyePACS dataset has been used which consists of 88,702 images, to reconstruct the input image. After optimization of SSMD-UNet for reconstructing task, we primarily used FGADR dataset to train the supervised path of SSMD-UNet for the segmentation of HEs, MAs, EXs, and SEs. The dataset is divided into three sets of 70% (1290), 5% (92) and 25% (460 images) for training, validation and testing, respectively.

We train four models, each optimized for its corresponding lesion. In order to train the model for HE detection, HE is considered as primary tasks while MA, EX and SE are considered as auxiliary tasks with the unsupervised reconstruction. Figure 5 shows the learning curves of each individual SSMD-UNet trained for the segmentation of EX, MA, SE, and HE lesions. The learning curve is plotted against the combined loss (${\mathcal {L}}_{\text {SSMD-UNet}}$) defined in Eq. (2).

The models are trained with the batch size of 16 using NVIDIA RTX 2090 GPU and Intel Core-i5 CPU, where we used the stochastic gradient descent (SGD) as optimizer with a learning rate of 0.0001. After each convolution layer, we applied batch normalization to achieve a stable distribution of activation values. The batch normalization layer was employed prior to the non-linearity layer. We utilized a non-linear activation function known as a rectified linear unit (ReLU) because it offers better performance related to hyperbolic tangent and leaky ReLU during validation. The structure of an encoder and the decoders are the same, however, the transposed convolution layers replaced the convolutional layers.

### Evaluation parameters

We employed five widely used metrics to evaluate the segmentation performance, such as, area under the curve of receiver operating characteristic (AUC-ROC), dice similarity coefficient, area under the curve of precision-recall (AUC-PR), mean absolute error (MAE), and sensitivity. We use the *Sigmoid* function in our evaluation as the final prediction $S_{p}$. Thus, we measure the similarity/dissimilarity between the pixel-level segmentation ground-truth *G*, and the final prediction map, which can be defined as follows:

#### Dice similarity coefficient

The dice similarity coefficient (DSC) is extensively used parameter defined in Eq. (5) to evaluate the degree of overlap of predicted segment ($S_{Pred}$) with ground truth segment ($S_{gt}$)^53^. The DSC values range between [0,1], where 1 and 0 represent complete overlap and no overlap, respectively.

5 $$\begin{aligned} DSC = \frac{2*S_{Pred}\cap S_{gt}}{S_{Pred} \, \cup \, S_{gt}} \end{aligned}$$

#### AUC-ROC

It relates the true positive rate versus false positive rate, in other words, compares sensitivity vs (1 − specificity). The higher the AUC-ROC, the bigger the difference between true negatives and true positives.

#### AUC-PR

This curve plot the positive predictive value in comparison with the true positive rate. The main focus of this metric is on the positive class and is unconcerned with the true negatives. Consequently, PR is more suitable than ROC, especially when the data is imbalanced.

#### Mean absolute error (MAE)

This metric calculates the pixel-wise error between $S_p$ and *G*, and can be defined as follows:

6 $$\begin{aligned} MAE = \frac{1}{w \times h} {\sum _{x}^{w}\sum _{y}^{h} |S_{p}(x,y) - G(x,y)|} \end{aligned}$$

#### Sensitivity

The classification of pixels performance and correctness of the segmentation area are measured by the sensitivity (SEN), as define below:

7 $$\begin{aligned} SEN = \frac{S_{Pred}\cap S_{gt}}{S_{gt} \, } \end{aligned}$$

## Results and discussion

We have carried-out multiple experiments on two publicly available datasets (i.e FGADR^5^ and IDRiD^9^) to evaluate the effectiveness of our proposed model. In this section, we emphasize five aspects of our model: (1) we quantify the overall performance of our model; (2) we elaborate on the effect of auxiliary tasks on enhancing the primary task performance; (3) we quantify the impact of using additional data; (4) we analyze the visual analysis and; (5) and eventually analyze the robustness analysis.

### Overall performance

We evaluate the overall performance of the proposed technique using the evaluation matrices such as dice score, AUC-ROC, AUC-PR, and MAE, as described in section evaluation paramets. We utilize FGADR^5^ dataset to analyze the performance of the proposed Multi-Decoder UNet architecture with semi-supervised learning (i.e, SSMD-UNet). Also, to expand our comparison, we implemented the proposed model without semi-supervised learning (SSL); utilized only labelled data for training. Table 1 provides the quantitative results of these experiments, where we compare our scheme with the existing state-of-the-art segmentation models. The experimental results illustrate that the proposed framework for diabetic retinopathy segmentation provides improved performance as compared to previous works.

**Table 1:** Quantitative results of deep learning-based lesion segmentation models on FGADR dataset.

Methods	MA				HE				EX				SE			
	Dice	ROC	PR	MAE	DICE	ROC	PR	MAE	Dice	ROC	PR	MAE	Dice	ROC	PR	MAE
FCN-8s^54^	0.468	0.925	0.363	0.006	0.509	0.962	0.606	0.011	0.586	0.981	0.686	0.009	0.637	0.963	0.642	0.005
DL_V3+ (s = 8)^55^	0.482	0.934	0.364	0.007	0.55	0.973	0.619	0.01	0.602	0.977	0.702	0.009	0.648	0.967	0.659	0.004
UNet-CL^56^ (semi-supervised)	0.166	–	–	–	0.365	–	–	–	0.382	–	–	–	0.475	–	–	–
U-Net^51^	0.521	0.927	0.382	0.005	0.57	0.967	0.643	0.011	0.607	0.982	0.726	0.009	0.655	0.977	0.683	0.003
Multi-class U-Net	0.515	0.923	0.389	0.005	0.547	0.967	0.647	0.01	0.618	0.982	0.731	0.01	0.649	0.976	0.685	0.004
Attention U-Net^57^	0.536	0.942	0.435	0.006	0.576	0.974	0.678	0.009	0.637	0.984	0.762	0.007	0.689	0.98	0.712	0.003
Gated U-Net^58^	0.529	0.945	0.441	0.006	0.58	0.978	0.682	0.009	0.638	0.983	0.764	0.007	0.685	0.982	0.716	0.003
Dense U-Net^59^	0.559	**0**.**959**	0.469	**0**.**004**	0.617	**0**.**981**	0.697	0.007	0.649	0.978	0.775	0.008	0.723	0.985	0.726	**0**.**002**
U-Net++^34^	0.533	0.937	0.453	0.005	0.597	0.974	0.689	0.009	0.644	0.98	0.771	0.008	0.719	0.984	0.722	0.003
MD-UNet	0.548	0.915	0.524	0.005	0.611	0.958	0.724	0.006	0.635	0.972	0.691	0.008	0.724	0.978	0.756	0.003
**SSMD-UNet (Ours)**	**0**.**579**	0.951	**0**.**646**	**0**.**004**	**0**.**653**	0.967	**0**.**824**	**0**.**004**	**0**.**662**	**0**.**988**	**0**.**790**	**0**.**0035**	**0**.**751**	**0**.**989**	**0**.**867**	0.003

The best results are illustrated in bold.

The results illustrate that our proposed model outperforms the current state-of-the-art segmentation approaches. The main reason for performance improvement is because of two factors: (1) the incorporation of MTL and; (2) SSL where we employ additional data of EyePACS dataset (unlabeled data) which is exploited in the unsupervised phase. The proposed model uses the encoding branch that plays an important role in enhancing the learning ability of the networks, it helps to extract latent representation which further eases the segmentation of the main task. Even without semi-supervised learning, our results are competitive which exhibits the effectiveness of MTL.

In contrast to traditional UNet and other deep learning-based lesion segmentation models, our proposed model employs multiple decoders within a multitask learning framework. This allows our network to concurrently learn a shared representation for multiple tasks, which enhances the system’s generalization as shown in Table 2. Additionally, our model is trained using a semi-supervised approach to effectively utilize the abundant unlabeled data available, resulting in improved performance as demonstrated in Fig. 2. This is not achievable using the conventional UNet architecture.

Furthermore, as depicted in Table 3, our proposed model outperforms other deep learning models for several reasons. For instance, the IDRiD dataset contains a limited number of labeled samples, and training deep learning-based lesion segmentation models typically requires a vast amount of labeled data. In Table 3, UNet, DeepLabV3+, and FCN are trained using only the limited samples, specifically 54 samples, without incorporating unlabeled data or a multi-decoder approach. Consequently, their results are significantly lower compared to our proposed model. The impact of each component is analyzed in the subsequent subsections.

**Table 2:** Performance evaluation of the proposed model on IDRiD and FGADR dataset.

Method	IDRiD		FGADR	
	Accuracy	IoU	Accuracy	IoU
SSMD-UNet	0.926	0.847	0.901	0.809

**Table 3:** Quantitative results of deep learning-based lesion models on IDRiD dataset.

Methods	MA			HE			EX			SE		
	Dice	ROC	PR	Dice	ROC	PR	Dice	ROC	PR	Dice	ROC	PR
UNet	0.225	0.431	0.265	0.251	0.462	0.324	0.297	0.493	0.364	0.342	0.483	0.349
DeepLabV3+	0.366	0.521	0.354	0.386	0.541	0.459	0.401	0.591	0.388	0.413	0.621	0.432
FCN	0.447	0.561	0.493	0.453	0.571	0.499	0.459	0.638	0.452	0.461	0.623	0.476
**SSMD-UNet**	**0**.**735**	**0**.**668**	**0**.**584**	**0**.**873**	**0**.**691**	**0**.**781**	**0**.**917**	**0**.**812**	**0**.**899**	**0**.**884**	**0**.**813**	**0**.**752**

The best results are illustrated in bold.

### Visual analysis

To further expand the comparison, we also visualize the results of a fully supervised version of the proposed model, i.e., MD-UNet, which may help to analyze the significance of SSL.

**Figure 6:**
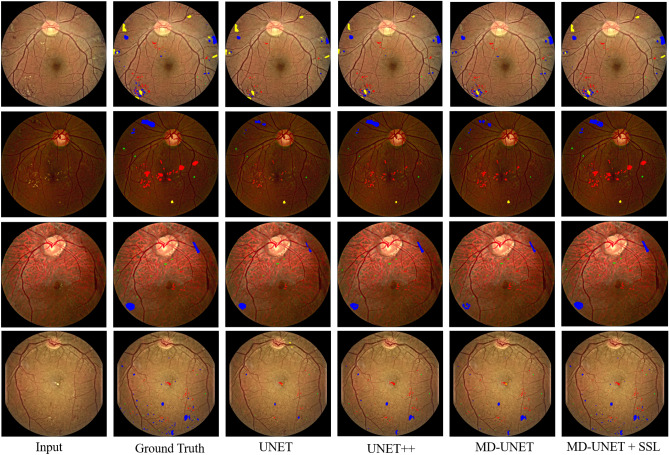
Segmentation results of FGADR dataset for MAs, HEs, EXs and SEs diseases from UNet, UNet++, MD-UNet and SSMD-UNet has been shown. Where MA, HE, EX and SE diseases are represented with green, blue, red and yellow colors, respectively.

Figure 6 show the results of four diseases (i.e., HE, MA, EX and SE) for different networks which include UNet^51^, UNet++^34^, MD-UNet and SSMD-UNet along with ground truth. To better visualize the difference between the diseases, we used color-coding to present each disease: green, blue, red and yellow colors that represent MAs, HEs, EXs and SEs, respectively. We observe the UNet and UNet++ detects only partial regions that correspond to red and blue lesions. While our proposed SSMD-UNet strategy are more close to the ground truth as compare to UNet and UNet++. Thus, we can conclude that our proposed model is effective for lesion segmentation task. Additionally, as seen in Fig. 6 our proposed model enhances the performance for all the diseases. It can also be noted that the performance of proposed scheme remain consistent for all the diseases while, UNet and UNet++ failed to demonstrate consistent performance against all four diseases. The main reason for performance improvement is because of two factors: (1) the incorporation of MTL and; (2) SSL where we employ additional data of EyePACS dataset (unlabeled data) which is exploited in the unsupervised phase.

**Figure 7:**
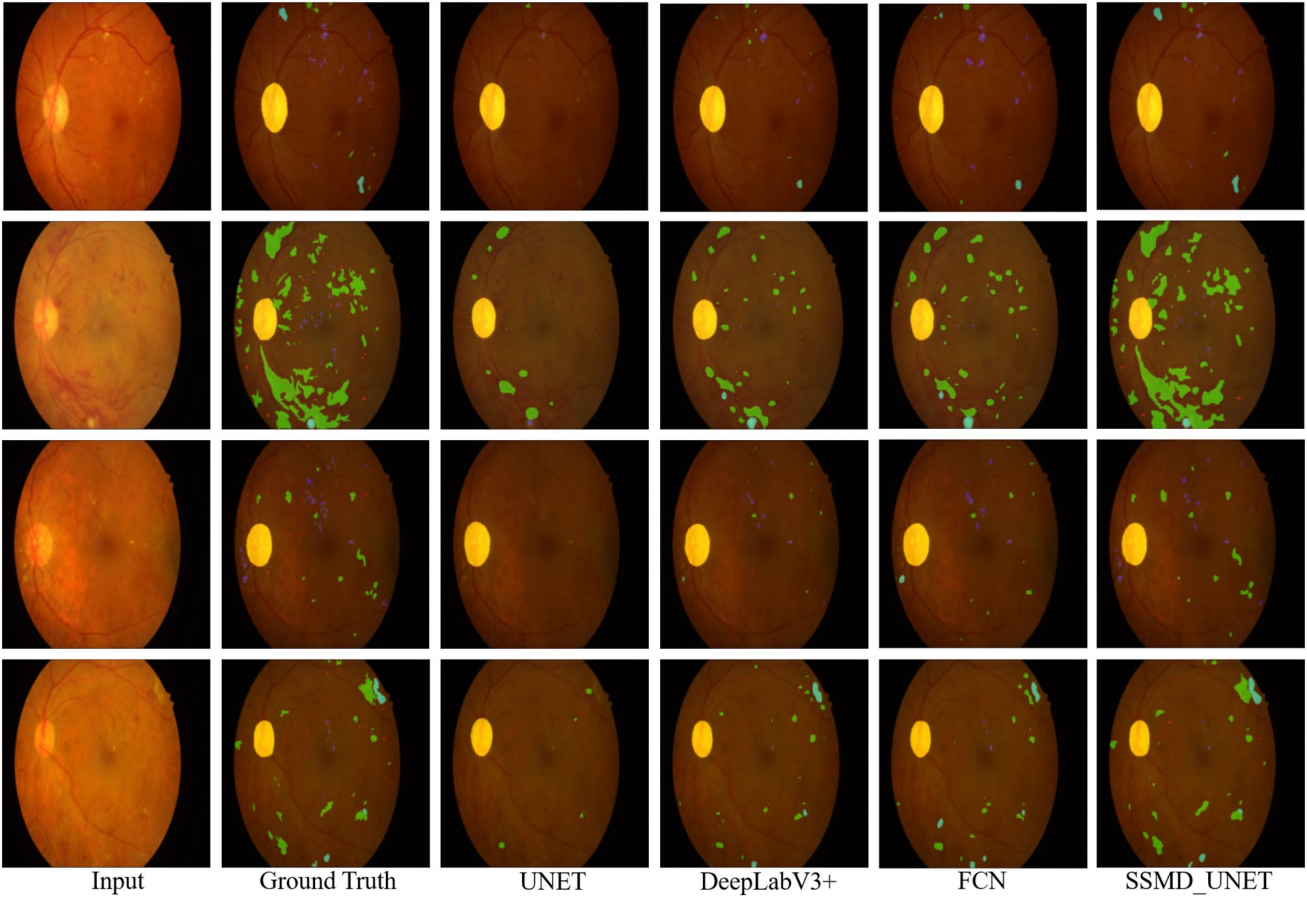
Segmentation results of IDRiD dataset for MAs, HEs, EXs and SEs diseases from UNet, DeepLabV3+, FCN, and SSMD-UNet has been shown. Where MA, HE, EX and SE diseases are represented with green, blue, red and yellow colors, respectively.

To better illustrate the effect of our proposed model, we also visualized the results of different images from IDRiD dataset. Figure 7 compare the segmentation results with corresponding original images, ground truths, UNet, DeepLabV3+, FCN, and our proposed SSMD-UNet. We observe that the UNet is not detecting the red lesion in IDRiD dataset. While, the DeepLabV3+ and FCN partially detect each lesion. On the other hand, our proposed model provides efficient results as can be seen in Fig. 7. The main reason for UNet, DeepLabV3+, and FCN low results are limited data.

### Effect of auxiliary tasks on primary task

**Figure 8:**
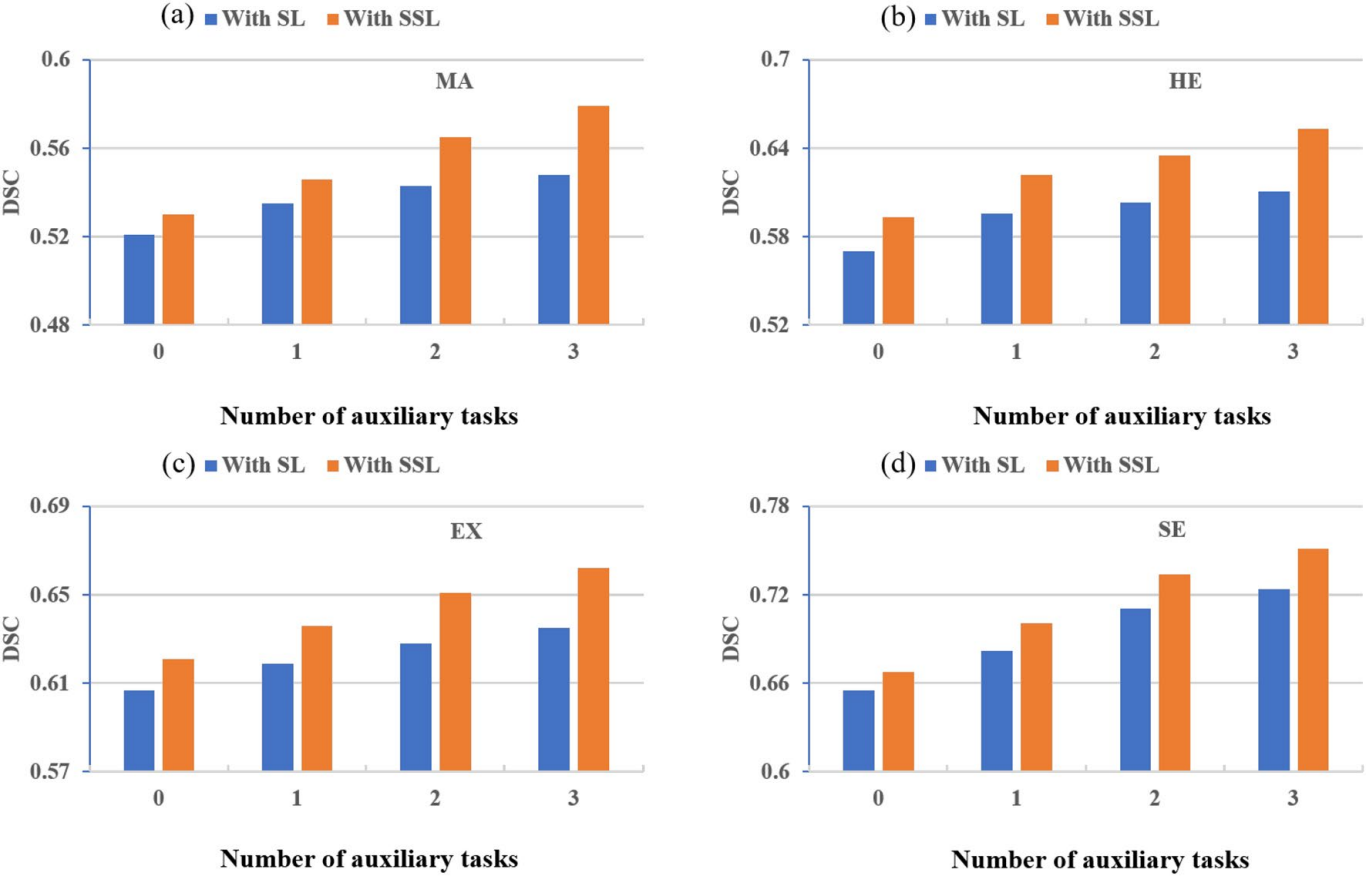
Illustrate the (**a**) MA (**b**) HE (**c**) EX and (**d**) SE results of proposed MD-Unet without SSL and with SSL Auxiliary tasks.

To investigate the effect of addition of the auxiliary tasks, we perform experiments with four different settings, i.e., without any auxiliary task, with 1, 2, and 3 auxiliary tasks. We also perform experiments with and without the incorporation of SSL which helps to better understand the effect of auxiliary tasks. The results have been illustrated in Fig. 8, where SL represents with supervised learning and SSL represents with semi-supervised learning settings while, 0 on x-axis correspond to the experiments without any auxiliary task, and 1, 2, and 3 shows the respective auxiliary tasks.

The results suggests that the incorporation of auxiliary tasks remarkably enhances the performance of primary tasks for each disease (i.e., HEs, MAs, EXs, and SEs). For each disease we obtain almost similar trend, notably, when we add a single auxiliary task, a significant improvement in the performance is noticed likewise, adding the second task, performance further improves. However, after adding the third auxiliary task, only a slight improvement in the performance is witnessed. It is also noticeable that the exploitation of semi-supervised learning further helps to capitalize the effect of auxiliary tasks. Without the application of the SSL scheme; using no unlabelled samples, we trained our MD-UNET model, we can still get the improvement in the results in terms of dice score. However, the improvement is much lesser than the SSL settings, where we utilize 88,702 additional images from EyePACS dataset.

The results demonstrates that the addition of auxiliary tasks enhances the generalisation of latent representation generated by the encoder, which subsequently eases the decoder of the primary task to segment the relevant lesion. We also noted that while adding the auxiliary task, initially performance improves drastically, however beyond two tasks we observe a plateauing effect. This is a critical observation that may lead the researchers to choose the optimal number of auxiliary tasks.

**Figure 9:**
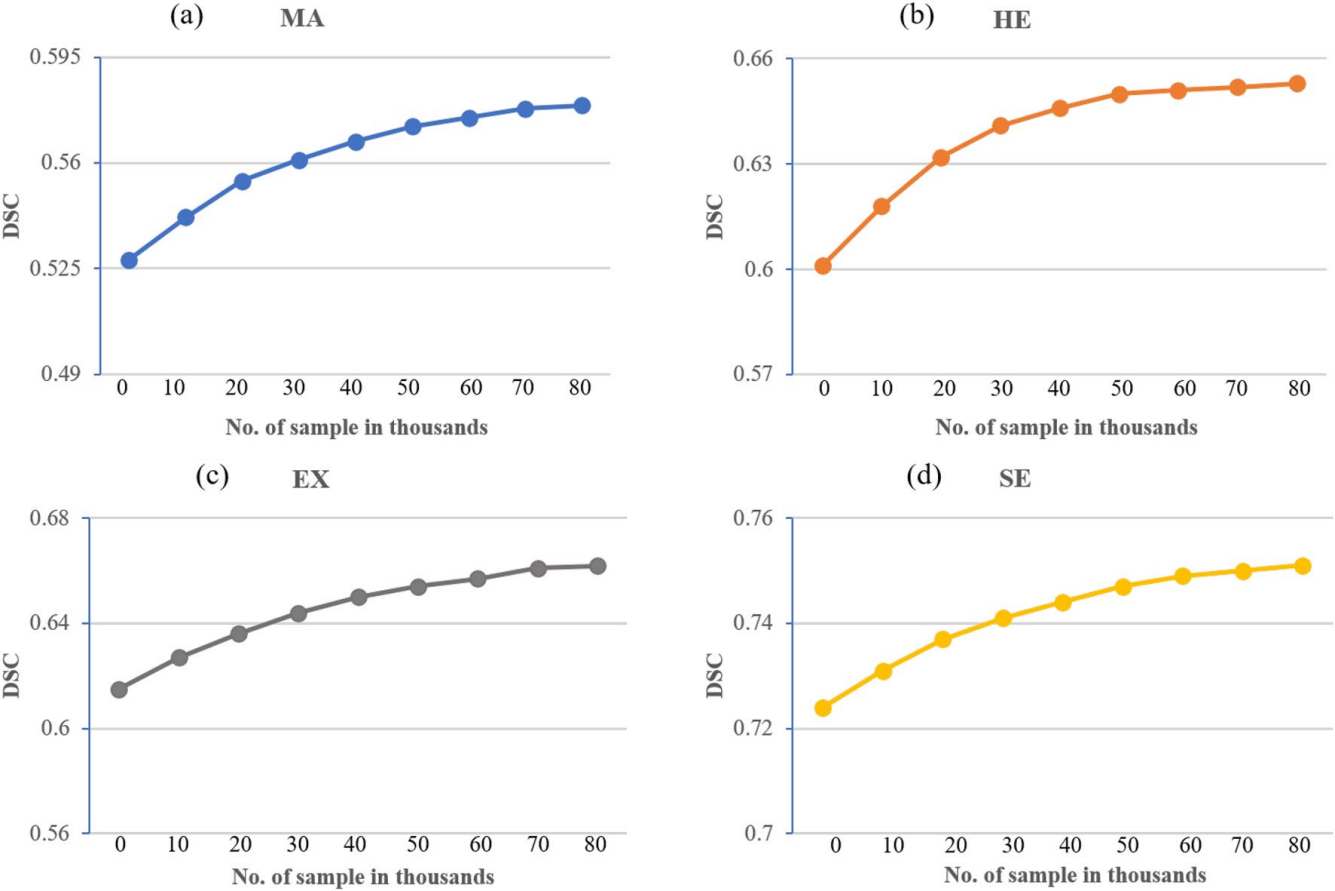
Effect of additional data on the performance of the system (**a**) MAs (**b**) HEs (**c**) EXs and (**d**) SEs dice score.

### Effect of using additional data

In this section, we further analysed the impact of incorporation of additional data on the performance of a primary task. We used additional unlabelled data during the unsupervised phase, where we train the network only for reconstruction tasks. We assess the effectiveness of additional data where we perform experiments by training the networks with different amounts of data and by following the same training strategy as mentioned in “Training strategy”. To further expand our analysis, we also experiment without using additional data; only multitask learning in the supervised phase is performed. Figure 9 demonstrates the results of our experiments for each disease in terms of dice score. The results depict the inclusion of additional data enormously improves the performance for primary tasks for each disease. As we increase the amount of data, performance for primary task also improves in each disease. However, the improvement is not consistent in each disease and follows a different pattern. Particularly, in the case of MAs and HEs, a drastic improvement in performance is observed till the addition of 40,000 images which is different for other diseases (i.e., EXs and SEs). Here we also notice that for each disease the performance drastically improves till a certain point after which it still improves but in a very gradual manner. This indicates that the encoder branch learns the meaningful information during the unsupervised phase and this learning improves when more data is provided. However, after a certain level, the incorporation of additional data does not significantly improve the results.

**Table 4:** Cross-dataset evaluation results for lesion segmentation on IDRiD dataset.

Method	% of IDRiD data used for training	SE/%	AUPR/%	AUC/%	DICE/%
Hemorrhage detection/segmentation					
Yan et al.^60^	66	**–**	70.3	**–**	–
Guo et al.^27^	66	**–**	–	63.74	–
Xu et al.^61^	66	73.42	–	–	84.50
** MD-UNet**	0	68.19	71.2	66.50	74.21
** SSMD-UNet**	0	70.35	74.8	68.23	79.10
** SSMD-UNet**	20	**74**.**87**	**78**.**14**	**69**.**15**	**87**.**35**
Microaneurysms detection/segmentation					
Xue et al.^11^	**–**	76.4	–	–	–
Yan et al.^60^	67	–	52.5	–	–
Guo et al.^27^	67	–	–	46.27	–
Sarhan et al.^62^	67	28.07	41.96	–	–
Xu et al.^61^	67	59.33	–	–	71.88
** MD-UNet**	0	62.92	50.91	62.89	67.23
** SSMD-UNet**	0	68.61	54.35	64.13	70.54
** SSMD-UNet**	20	**77**.**14**	**58**.**42**	**66**.**85**	**73**.**52**
Soft exudate detection/segmentation					
Yan et al.^60^	65	–	67.9	–	–
Guo et al.^27^	65	–	–	71.13	–
Xu et al.^61^	65	79.33	–	–	88.12
** MD-UNet**	0	71.46	69.88	76.24	81.75
** SSMD-UNet**	0	76.76	72.14	77.92	84.68
** SSMD-UNet**	20	**80**.**35**	**75**.**21**	**81**.**36**	**88**.**45**
Hard exudate detection/segmentation					
Xue et al.^11^	**–**	77.9	–	–	–
Yan et al.^60^	67	–	88.9	–	–
Guo et al.^27^	67	–	–	79.45	–
Xue et al.^61^	67	87.55	–	–	91.38
** MD-UNet**	0	77.56	76.81	74.65	83.47
** SSMD-UNet**	0	83.21	80.14	78.21	86.29
** SSMD-UNet**	2%	**88**.**72**	**89**.**94**	**81**.**3**	**91**.**75**

The best results are illustrated in bold.

### Robustness analysis

To evaluate the robustness of propose scheme, we performed cross-dataset validation. We trained our model with EyePACS dataset in unsupervised phase and use whole FGADR^5^ dataset during the supervised phase. To verify the generalisation ability of proposed scheme, we use the IDRiD dataset for evaluation without training the models on IDRiD. However, to better compare our results with previous works, we retrained the proposed SSMD-UNet scheme by adding 20% of IDRiD into the training dataset. In Table 4, the results have been compared with other studies that have also utilized IDRiD dataset for training the models. The cross-data performance has been evaluated against various parameters listed in “ Evaluation parameters” along with another parameter (i.e., sensitivity) for better transparency. The results demonstrate that the proposed scheme achieve better performances in comparison with the previous technique. These results also suggest that the incorporation of a large EyePACS dataset during the unsupervised phase, enables the encoder block to learn the meaningful information in a generalized manner. Subsequently, the model further refine its learning in the supervised phase which lead to a highly robust solution.

## Conclusions and future works

We propose a novel semi-supervised learning based Multi-Decoder UNet for the segmentation of DR lesions including HEs, MAs, EXs, and SEs using fundus images. Our proposed architecture consists of single encoding and five decoding blocks (i.e., one for reconstruction and four for segmentation tasks). Specifically, we trained our model in a semi-supervised way to utilise the readily available unlabelled data to improve the generalisation of model that subsequently leads to an improved performance for each disease. The proposed scheme has been extensively evaluated on two datasets including FGADR and IDRiD. The results illustrate that our scheme has outperformed the state-of-the-art techniques and also has demonstrated significant robustness while cross-dataset analysis. Future work includes the incorporation of adversarial learning to further improve the representation learning of encoder branch by enforcing the desired distribution which may help the classification.

## Data Availability

The IDRiD dataset is publicly available online at: https://ieee-dataport.org/open-access/indian-diabetic-retinopathy-image-dataset-idrid and Kaggle-EyePACS dataset is available online in the repository at: https://www.kaggle.com/c/diabetic-retinopathy-detection, while the FGADR dataset is available from the corresponding author on reasonable request.
